# Assessing Menstrual Changes Among Young Indian Females Post-SARS-CoV-2 Vaccination

**DOI:** 10.7759/cureus.50025

**Published:** 2023-12-06

**Authors:** Archita Tandon, Naveen Kumar, Sunita Aggarwal, Yogita Anjana, Mohinder P Sachdeva, Vipin Gupta, Geeta Trilok-Kumar

**Affiliations:** 1 Anthropology, University of Delhi, New Delhi, IND; 2 Biotechnology and Research, Sir Ganga Ram Hospital, New Delhi, IND; 3 Microbiology, Institute of Home Economics, University of Delhi, New Delhi, IND; 4 Food and Nutrition, Institute of Home Economics, University of Delhi, New Delhi, IND; 5 Epidemiology and Public Health, Institute of Home Economics, University of Delhi, New Delhi, IND; 6 Nutrition, Trivedi School of BioSciences, Ashoka University, Haryana, IND

**Keywords:** covid-19 india, menstrual pain, menstrual cycle irregularity, sars-cov-2 vaccines, sars-cov-2

## Abstract

Background

The rollout of severe acute respiratory syndrome coronavirus 2 (SARS-CoV-2) vaccines has significantly enhanced immunity against coronavirus disease 2019 (COVID-19), leading to a reduction in the severity of illness, hospitalizations, and deaths. While various side effects of the vaccine have been reported, its impact on the menstrual cycle remains unclear.

Methods

We conducted a cross-sectional study involving university students who had received either partial or full vaccination against SARS-CoV-2. Data was gathered through a questionnaire designed to assess the relationship between menstrual changes and the SARS-CoV-2 vaccination.

Results

A total of 773 participants, with a mean age of 20.6 ± 1.7 years, were included in this study. The participants reported a significant increase in the irregularity of the menstrual cycle. We observed a slight increase in the length of the menstrual cycle, from 30.0 ± 4.0 days (pre-vaccination) to 30.5 ± 5.6 days (post-vaccination), which was statistically significant (p<0.001). The duration of menstruation also increased, from 4.9 ± 1.7 days (pre-vaccination) to 5.0 ± 1.7 days (post-vaccination). However, this increase in menstrual length due to vaccination was not statistically significant (p = 0.898). Notably, there was a significant increase in pain reported by the participants after receiving the SARS-CoV-2 vaccine (p = 0.004).

Conclusion

The SARS-CoV-2 vaccination significantly impacted the regularity of the menstrual cycle, length of the menstrual cycle, and pain during menstruation, though temporarily. Our study found no significant differences in menstrual changes or the type of vaccine administered (Covishield and Covaxin).

## Introduction

Vaccination programs have played a pivotal role in bolstering immunity against severe acute respiratory syndrome coronavirus 2 (SARS-CoV-2) infections [1-3]. These vaccines, developed through various immunologically targeted techniques, were swiftly authorized for emergency use in 2020. Health authorities worldwide diligently informed the public about the common local and systemic side effects associated with these vaccines, including sore arms, myalgia, fever, and fatigue [4-6]. However, changes in the menstrual cycle were notably absent from the list of recognized side effects of SARS-CoV-2 vaccines [7,8].

The emergence of self-reported menstruation-related issues on social media platforms prompted researchers across the globe to scrutinize the potential impact of the SARS-CoV-2 vaccination on menstrual changes [9,10]. In the absence of robust population-based evidence regarding the link between menstrual changes and SARS-CoV-2 vaccines in 2021, this study was initiated among Indian women, with data collection focused on university students.

During our data collection phase, cross-sectional and cohort studies were published, shedding light on an association between altered menstrual symptoms and SARS-CoV-2 vaccines. A US-based cohort study reported a modest change of less than one day in menstrual cycle length, while a study conducted in the Middle East and North Africa (MENA) region revealed that 66.3% of women associated menstrual changes with SARS-CoV-2 vaccination [10,11]. Furthermore, yellow card reports by healthcare workers in the United Kingdom (UK) documented post-vaccination menstrual changes in nearly 80% of individuals, with these changes linked to the female body's immune response to the SARS-CoV-2 vaccine [12]. Therefore, the primary aim of this study is to investigate the association between SARS-CoV-2 vaccination and menstrual changes, with the hypothesis that SARS-CoV-2 vaccination impacts menstruation.

## Materials and methods

Study design

A cross-sectional study was conducted among students of the University of Delhi between November 2021 and February 2022. Data was collected through a questionnaire designed to retrospectively assess the association between menstrual changes and the SARS-CoV-2 vaccination. Female participants aged 18 to 25 years who had received either partial or full vaccination with either of the two available vaccines in India (Covishield, ChAdOx1 nCoV-190 by Oxford/AstraZeneca, or Covaxin BBV152 by Bharat Biotech) were included in this study. Exclusion criteria encompassed women vaccinated with vaccines other than Covishield or Covaxin; those with polycystic ovarian disorder (PCOD), polycystic ovarian syndrome (PCOS), or other clinically diagnosed existing menstrual disorders; and pregnant or lactating women.

A small pilot study was conducted in October 2021 among college students at the University of Delhi, with 60 participants. Based on the responses and results of the pilot study, the questionnaire was modified, and the participants from the pilot study were not included in the final study. The sample size was calculated as 600 using the formula: sample size = Z2*(p) *(1-p)/c2, where Z = Z value (1.96 for a 95% confidence level), p = percentage choosing an option, and c = confidence interval (±4).

Data collection and analysis

Data was collected using a questionnaire distributed to students at the University of Delhi. Participants completed the questionnaire during university hours. Each participant was provided with an explanation of the study's purpose along with an information sheet and asked to sign the consent form if they were willing to participate. Any questions from participants were addressed during the enrollment process.

Statistical Package for Social Sciences (SPSS) Statistics version 18 (IBM Corp., Armonk, NY, USA) was used to compare participants' responses before and after vaccination using a chi-square test considered significant at p <0.05 to analyze the impact of the vaccine on the following aspects: (i) regularity of the menstrual cycle, (ii) length of the menstrual cycle, (iii) length of menstruation, (iv) amount of bleeding during menstruation, and (v) pain experienced during menstruation. Additionally, the impact of the type of vaccine administered (Covaxin and Covishield) was compared.

## Results

**Figure 1:**
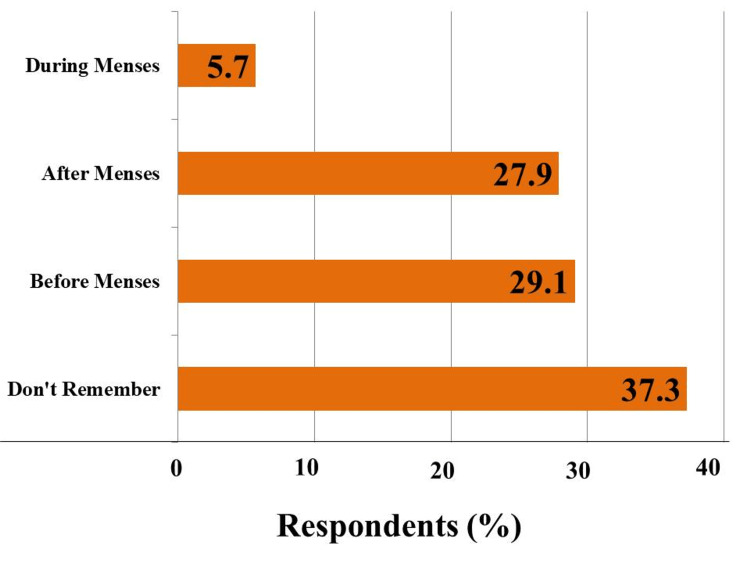
Time of vaccination

Out of the initial 954 participants, 181 individuals were excluded from the study due to incomplete information or if they were vaccinated with a vaccine other than Covaxin or Covishield. Consequently, a total of 773 participants were included in this study for further analysis. Table 1 provides an overview of the descriptive characteristics of the study participants. More than half of the participants were undergraduates, with a mean age of 20.6 ± 1.7 years. A majority (77.6%) of the participants had received the Covishield vaccine, and 80% of the participants were fully vaccinated. Approximately 10% of the participants expressed hesitation in receiving the vaccine, primarily due to concerns about potential side effects, with 2.9% of these participants specifically fearing its impact on menstruation. Around 18% of the participants reported experiencing changes in their menstrual cycle after the first dose, 5.9% after the second dose, and 12.8% after both doses of the SARS-CoV-2 vaccine. More than half of the participants received their vaccination either before or after menstruation, and approximately a third (37.3%) could not recall the exact timing of their vaccination (Figure 1).

**Table 1:** Descriptive characteristics of the study participants (n=773)

Variables	Frequency	Percentage
Age, Years (Mean±SD)	20.6±1.7	
Education		
Undergraduate	443	57.3
Graduate	309	40.0
Postgraduate	21	2.7
Vaccine type		
Covaxin	173	22.4
Covishield	600	77.6
Number of doses		
1^st^ dose	161	20.8
2^nd^ dose	612	79.2
Hesitant to take the vaccine?		
Yes	77	10.0
No	696	90.0
Place of 1^st^ dose		
Government centre	688	89.0
Private centre	85	11.0
Place of 2^nd^ dose		
Government centre	563	92.0
Private centre	49	8.0
Abnormalities experienced in the menstrual cycle post-vaccination		
After both doses	99	12.8
First dose	136	17.5
Second dose	46	5.9
None	494	63.7

Association of SARS-CoV-2 vaccine with menstrual cycle regularity

Participants were asked to provide information about the regularity of their menstrual cycle, both before and after vaccination. A significant increase in the number of participants reporting irregularities in their menstrual cycle after receiving the SARS-CoV-2 vaccine was observed (p <0.001). However, we found that the type of vaccine administered (Covaxin and Covishield) was not associated with the irregularity of the menstrual cycle (p = 0.147), as indicated in Table 2.

**Table 2:** Regularity of menstrual cycle pre- and post-vaccination in correlation with the type of vaccine taken by the participants

Menstrual cycle	Pre-vaccination: n (%)	Post-vaccination: n (%)	p-value	Vaccine type: n (%)		p-value
				Covaxin	Covishield	
Regular	642 (83.1)	582 (75.3)	<0.001	123 (71.1)	459 (76.50)	0.147
Irregular	131 (16.9)	191 (24.7)		50 (28.90)	141 (23.50)	
Total	773 (100)	773 (100)		173 (100)	600 (100)	

Out of 773 participants, 49 were unable to recall the specificity of the irregularities in the menstrual cycle, i.e., the number of days menstruation was delayed or early. Therefore, out of 724 participants, nearly 68% reported a consistent, regular menstrual cycle. However, the remaining participants reported various irregularities, such as delayed or early menstruation occurring after vaccination, and some with delays of more than 20 days (as shown in Table 3).

**Table 3:** Distribution of irregularity in menstrual cycle with respect to the early or late onset of menstruation Of the participants, 49 were unable to recall irregularities, i.e., the number of days by which menstruation was delayed or early. Therefore, 724 participants are analyzed here.

Menstrual cycle	No. of days	Frequency: n (%)
Early	21 to 30	2 (0.3)
	11 to 20	8 (1.1)
	1 to 10	63 (8.7)
Regular	0	493 (68.1)
Delayed	1 to 10	117 (16.2)
	11 to 20	40 (5.5)
	21 to 30	1 (0.1)

Association of SARS-CoV-2 vaccine with menstrual cycle length

The mean length of the menstrual cycle before vaccination was 30.0 ± 4.0 days (n = 697), while after vaccination, it was 30.5 ± 5.6 days (n = 676). Participants experienced a mean increase of 0.5 days in the length of their menstrual cycle. This change in the length of the menstrual cycle post-vaccination was statistically significant (p < 0.001). Similarly, for menstrual cycle regularity, we found no significant difference in the association between the type of vaccine administered and the length of the menstrual cycle (p = 0.909), as presented in Table 4.

**Table 4:** Length of the menstrual cycle pre- and post-vaccination in correlation with the type of vaccine The variation in the number of participants is because the participants were unable to recall the correct details.

Length of menstrual cycle (days)	Pre-vaccination: n (%)	Post-vaccination: n (%)	p-value	Vaccine type: n (%)		p-value
				Covaxin	Covishield	
15-25	0 (0)	35 (5.2)	<0.001	7 (4.5)	28 (5.4)	0.909
26-35	639 (91.9)	550 (81.6)		126 (81.8)	424 (81.5)	
≥36	56 (8.1)	89 (13.2)		21 (13.7)	68 (13.1)	
Total	695 (100)	674 (100)		154 (100)	520 (100)	

Association of SARS-CoV-2 vaccine with menstrual duration

Participants reported a slight increase in the length of menstruation following the SARS-CoV-2 vaccination. The mean duration of menstruation prior to vaccination was 4.9 ± 1.7 days, and this increased to a mean duration of 5.0 ± 1.7 days after vaccination. However, this increase in menstrual length due to vaccination was not found to be statistically significant (p = 0.898). Additionally, we found no significant difference in the association between the type of vaccines administered and the duration of menstruation, as outlined in Table 5.

**Table 5:** Duration of menstruation pre- and post-vaccination in correlation with the type of vaccine

Duration of menstruation (days)	Pre-vaccination: n (%)	Post-vaccination: n (%)	p-value	Vaccine type: n (%)		p-value
				Covaxin	Covishield	
1-3	90 (11.6)	95 (12.3)	0.898	21 (12.1)	74 (12.3)	0.400
4-6	596 (77.1)	585 (75.7)		133 (76.9)	452 (75.3)	
7-10	76 (9.8)	83 (10.7)		19 (11.0)	64 (10.7)	
>10	11 (1.5)	10 (1.3)		0 (0)	10 (1.7)	
Total	773 (100)	773 (100)		173 (100)	600 (100)	

Association of SARS-CoV-2 vaccine with menstrual flow (volume)based on sanitary pad-usage

To evaluate menstrual flow, we assessed the number of sanitary pads used per day. Our findings indicated a slight increase in the number of sanitary pads used by participants following the SARS-CoV-2 vaccination. However, this increase did not reach statistical significance (p = 0.349). Moreover, we found no significant association between the type of vaccine administered and menstrual flow post-vaccination, as detailed in Table 6.

**Table 6:** Menstrual flow (volume) pre- and post-vaccination in correlation with the type of vaccine *In cases where participants did not use menstrual pads, other methods such as tampons or menstrual cups were employed, and the results were categorized as 'not applicable'.

No. of sanitary pads used per day	Pre-vaccination: n (%)	Post-vaccination: n (%)	p-value	Vaccine type: n (%)		p-value
				Covaxin	Covishield	
1-2	250 (32.3)	232 (30.0)	0.349	52 (30.1)	180 (30.0)	0.907
3-4	434 (56.1)	425 (55.1)		97 (56.1)	326 (54.3)	
5-6	47 (6.1)	73 (9.4)		14 (8.1)	59 (9.8)	
7-8	16 (2.1)	14 (1.8)		3 (1.7)	13 (2.2)	
9-10	12 (1.6)	13 (1.7)		3 (1.7)	10 (1.7)	
>10	7 (0.9)	8 (1.0)		1 (0.6)	7 (1.2)	
Not applicable*	7 (0.9)	8 (1.0)		3 (1.7)	5 (0.8)	
Total	773 (100)	773 (100)		173 (100)	600 (100)	

Association of SARS-CoV-2 vaccine with menstrual pain

The pain experienced by participants was assessed using a pain scale ranging from 1 to 10, where 1 represented minimal or no pain and 10 indicated maximum pain. Prior to vaccination, participants reported a mean pain score of 4.5 ± 2.3. However, post-vaccination, this mean pain score increased to 5.3 ± 2.4. Significantly, our study observed a notable increase in pain among the participants following the SARS-CoV-2 vaccination (p = 0.004). Additionally, we found no statistically significant difference between the increase in pain and the type of vaccine administered, as demonstrated in Table 7.

**Table 7:** Pain experienced during menstruation pre- and post-vaccination in correlation with the type of vaccine

Pain scale	Pre-vaccination: n (%)	Post-vaccination: n (%)	p-value	Vaccine type: n (%)		p-value
				Covaxin	Covishield	
1-4	353 (45.7)	277 (35.8)	<0.001	59 (34.1)	217 (36.2)	0.519
5-7	311 (40.2)	337 (43.6)		82 (47.4)	256 (42.6)	
8-10	109 (14.1)	159 (20.6)		32 (18.5)	127 (21.2)	
Total	773 (100)	773 (100)		173 (100)	600 (100)	

Restoration of normal menstrual cycle

**Figure 2:**
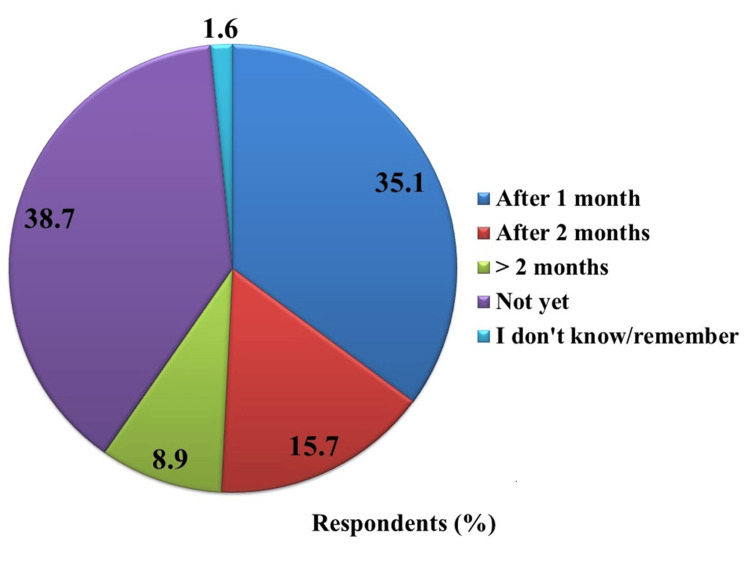
Distribution of time of return to normal menstrual cycle (n=191)

The menstrual changes observed after vaccination did not appear to have a long-term impact, as they were typically restored to normal within one to three months. Among the participants, a quarter (n = 191) reported experiencing abnormalities in their menstrual cycle. Of these participants, 35.1% reported their menstrual cycle returning to normal within one month after vaccination. Another 15.7 mentioned the return to a regular menstrual cycle within two months, while 8.9% of participants required more than two months for the restoration. Approximately 38.7% of the participants indicated that their menstrual cycle had not returned to normal at the time of data collection, and 1.6% could not recall, as illustrated in Figure 2.

## Discussion

The global deployment of SARS-CoV-2 vaccines has been instrumental in enhancing immunity against COVID-19, significantly reducing the severity of illness, hospitalizations, and fatalities [1-3]. While rare instances of adverse effects, including allergic reactions, have been reported, the most common side effects after SARS-CoV-2 vaccination are mild and temporary, such as fever, headache, fatigue, and pain [8,13]. However, the potential impact of SARS-CoV-2 vaccines on the menstrual cycle was not initially documented as a recognized side effect, despite anecdotal reports from a subset of women who shared their experiences on social media [9,10,14,15].

This cross-sectional study was conducted to investigate the changes in the menstrual cycle post-SARS-CoV-2 vaccination by retrospectively gathering data from 773 female students at the University of Delhi. In India, the first two vaccines authorized for emergency use were Covishield and Covaxin, and despite administering more than 2 billion doses, there has been no official update on adverse drug reactions (ADRs) related to their impact on the menstrual cycle [16]. The menstrual cycle involves complex inflammatory responses in angiogenesis, tissue repair, endometrial proliferation, and remodeling, governed by cytokine and chemokine regulators in the female reproductive tract. Notably, the SARS-CoV-2 infection itself is a pro-inflammatory condition that can lead to immune exhaustion. Vaccination against SARS-CoV-2 generates neutralizing antibodies and can result in the production of endometrial leukocytes, potentially leading to instances of menstrual changes [17-19].

In our study, over 75% of the participants received the Covishield vaccine, and we found a significant association between the SARS-CoV-2 vaccination and both the length and regularity of the menstrual cycle. Our findings align with several reported studies on the impact of vaccination on the menstrual cycle. For instance, a cross-sectional study conducted through an online survey as part of the retrospective EVA project that studied the effect of vaccination against SARS-CoV-2 on the menstrual cycle reported that 38% of participants experienced delayed menstruation, while 32% reported early menstruation post-SARS-CoV-2 vaccination [20]. Another retrospective online study involving females aged 18 to 41 years also reported that 20.8% of participants experienced delayed menstruation and 21.4% experienced early menstruation, findings that mirror our own study [19]. Similarly, a study among Pakistani women aged 18 and above reported that one-tenth of the participants missed their menstrual cycle post-SARS-CoV-2 vaccination, further supporting our findings [21]. Our study also aligns with the findings of the Arizona COVID-19 cohort (CoVHORT), which also documented irregularities in the menstrual cycle in 43% of the participants [22]. In a cross-sectional study involving premenopausal Lebanese women, 20.4% of participants were found to have irregular menstrual cycles, consistent with the findings of our own study in this regard [23].

Several studies have sought to explain this post-vaccination amenorrhea by the energy expenditure involved in processes such as leukocyte activation of T-lymphocytes in the production of antibodies against COVID-19, which can induce a stress response affecting the hypothalamic-pituitary-gonadal axis and leading to amenorrhea [19,24,25]. Some studies also reported occurrences of inflammatory and autoimmune responses leading to hypophysitis, a condition associated with amenorrhea and hypogonadotropic hypogonadism, effects on hormonal structure [26], long-term endocrine-metabolic complications [27], and many different cases of pituitary and thyroid disorders following vaccination [28-30].

Regarding changes in the duration of menstruation, we observed a 0.5-day increase among vaccinated individuals, similar to the findings of a US cohort study and a cross-sectional study conducted in the MENA region [10,11]. Our study did not find any association between the type of vaccine administered and changes in the menstrual cycle, which contrasts with studies reporting differences in the impact of various vaccines on the menstrual cycle. For example, one study suggested that the Oxford/AstraZeneca vaccine had a greater effect on the menstrual cycle compared to other vaccines like Pfizer, Janssen, or Moderna [20]. However, our results align with those from the cross-sectional study in the MENA region [11]. Additionally, our study revealed a significant association between SARS-CoV-2 vaccination and menstrual pain, which is consistent with the findings reported by the EVA project [20] and CoVHORT [22]. Another cross-sectional online survey-based study from six Arab countries also reported an increase in menstrual pain post-vaccination [31].

Our study observed contradictory results to studies showing changes in menstrual flow post-vaccination, as some studies reported increased menstrual flow after vaccination [19,20,22,23,31,32]. The findings of our study suggest that the changes in the menstrual cycle are temporary and may be attributed to a prolonged immune response initiated by vaccination, potentially affecting the menstrual cycle temporarily, as reported by other authors [10,19,22,31,32].

This study serves as a crucial foundation for further research to elucidate the association between menstrual changes and SARS-CoV-2 vaccination. It is important to note that the present findings are based on a specific age group of menstruating females and cannot be generalized to all women. Future research is required to establish the cause-and-effect relationship and underlying physiological mechanisms governing these menstrual changes across a larger, more representative group of women. This study marks a pioneering effort among the Indian population, highlighting the association between SARS-CoV-2 vaccines and menstrual changes in terms of cycle length, regularity, and pain during menstruation. Subsequent studies are necessary to confirm both the short-term and long-term menstrual changes following the SARS-CoV-2 vaccination.

Strengths and limitations

The primary strength of our study is its substantial sample size, which provides robust data for analysis. This large sample size enhances the generalizability of our findings among the studied age group of menstruating females. However, our study does have certain limitations. Firstly, most of the responses are reliant on participants' recall, which introduces the possibility of recall bias due to self-reported data. Secondly, the cross-sectional study design restricts our ability to establish causal relationships. Longitudinal studies would be better suited for this purpose. The age group of the study participants does not encompass the entire menstruating Indian population, limiting the generalizability of our findings to a broader demographic. Moreover, the study's exclusion of participants with pre-existing menstrual disorders may underrepresent the impact of SARS-CoV-2 vaccines on this specific subgroup. Furthermore, our study did not consider the endocrinological consequences following vaccination, which could have provided a more comprehensive understanding of the underlying reasons behind the reported menstrual irregularities.

## Conclusions

Our study has revealed that the SARS-CoV-2 vaccines, Covishield and Covaxin, administered in India, have been associated with menstrual irregularities in females. Our analysis focused on five critical menstrual cycle characteristics, namely regularity of the menstrual cycle, length of menstrual cycle, length of menstruation, menstrual flow, and pain during menstruation. Out of these five parameters, regularity of the menstrual cycle, length of the menstrual cycle, and pain during menstruation demonstrated significant impacts resulting from the SARS-CoV-2 vaccination. However, it is crucial to note that these changes were temporary. Our study found no significant disparities in menstrual changes related to the type of vaccine administered, whether Covishield or Covaxin. Larger, multicentre, prospective, and cohort studies are warranted to further investigate and validate the impact of SARS-CoV-2 vaccines on menstrual health among females.
